# circ-PRKCB acts as a ceRNA to regulate p66Shc-mediated oxidative stress in intestinal ischemia/reperfusion

**DOI:** 10.7150/thno.44250

**Published:** 2020-08-29

**Authors:** Dongcheng Feng, Zhecheng Wang, Yan Zhao, Yang Li, Deshun Liu, Zhao Chen, Shili Ning, Yan Hu, Jihong Yao, Xiaofeng Tian

**Affiliations:** 1 Department of General Surgery, The Second Affiliated Hospital of Dalian Medical University, Dalian 116023, China.; 2 Department of General Surgery, The First Affiliated Hospital of Dalian Medical University, Dalian 116600, China.; 3 Department of Pharmacology, Dalian Medical University, Dalian 116044, China.; 4 Department of Pharmacy, The Second Affiliated Hospital of Dalian Medical University, Dalian 116023, China.

**Keywords:** intestinal ischemia/reperfusion, oxidative stress, p66Shc, miR-339-5p, circular RNA

## Abstract

**Background:** Oxidative stress has emerged as an essential factor in the pathogenesis of intestinal ischemia/reperfusion (I/R) injury. The adaptor protein p66Shc is a key regulator of reactive oxygen species (ROS) generation and a mediator of I/R damage in the intestine, but the upstream mechanisms that directly regulate p66Shc expression during intestinal I/R remain largely unknown. Recent studies have suggested that noncoding RNAs, such as circular RNAs (circRNAs), are important players in physiological and pathological processes based on their versatile regulatory roles in gene expression. The aim of this study was to elucidate the contribution of p66Shc to oxidative damage in intestinal I/R and to investigate the regulation of p66Shc by circRNA sponges.

**Methods:** Intestinal I/R was induced in mice via superior mesenteric artery (SMA) occlusion. A miR-339-5p agomir or circ-protein kinase C beta (PRKCB) siRNA was injected intravenously before I/R challenge. In addition, Caco-2 cells were subjected to hypoxia/reoxygenation (H/R) *in vitro* to simulate an *in vivo* I/R model.

**Results:** *In vitro*, p66Shc deficiency significantly reduced H/R-induced ROS overproduction by attenuating mitochondrial superoxide anion (O_2_^-^) levels, suppressing NADPH oxidase activity and enhancing antioxidant enzyme expression. Moreover, miR-339-5p was identified to directly regulate p66Shc expression in the intestine. Furthermore, we found that a circRNA transcribed from the PRKCB gene, named circ-PRKCB, acted as an endogenous miR-339-5p sponge to regulate p66Shc expression. circ-PRKCB silencing or miR-339-5p overexpression significantly downregulated p66Shc expression and attenuated oxidative stress levels and I/R injury *in vivo* and *in vitro*. Notably, the increased circ-PRKCB levels and decreased miR-339-5p levels associated with murine intestinal I/R were consistent with those in patients with intestinal infarction.

**Conclusions:** Our findings reveal a crucial role for the circ-PRKCB/miR-339-5p/p66Shc signaling pathway in regulating oxidative stress in the I/R intestine. This pathway may be a potential therapeutic target for intestinal I/R injury.

## Introduction

Intestinal ischemia/reperfusion (I/R) injury is well known as a critical clinical event that is secondary to mesenteric vascular occlusion, volvulus, trauma, shock states, and small bowel transplantation and can ultimately progress to multiple organ failure and often death due to local and systemic injuries 1-3. The factors contributing to intestinal I/R injury are quite complex. Recently, accumulating evidence has revealed that oxidative stress is crucially involved in the pathogenesis of intestinal I/R 4. Excessive reactive oxygen species (ROS) generation in intestinal epithelial cells can damage virtually every intracellular biomolecule and activate inflammatory and cell death cascades to exacerbate reperfusion injury 5, 6. Despite extensive research on antioxidant agents designed to reduce ROS levels, no pharmacological therapies have been demonstrated to improve clinical outcomes 7. Therefore, novel treatment approaches, specifically those targeting the complex redox signaling network, are urgently needed for intestinal I/R injury.

P66Shc, an isoform of the mammalian adaptor protein ShcA, has emerged as a key redox molecule responsible for intracellular ROS generation 8. Indeed, intracellular free radical levels are significantly reduced in cells lacking the p66Shc gene as well as in p66Shc-/- mouse models exposed to severe oxidative stress 9. Growing evidence has demonstrated that p66Shc-mediated oxidative stress contributes to numerous oxidation-related pathological conditions, including organ I/R injuries. Our previous study has shown that increased p66Shc expression is associated with gut injury after intestinal I/R 10. Moreover, inhibiting the p66Shc pathway by targeting posttranslational modification factors confers protection against intestinal I/R injury 11, 12. These findings suggest that p66Shc may be a promising candidate for therapeutic intervention. However, there is limited information about the upstream mechanisms that directly regulate p66Shc expression levels during intestinal I/R. This may represent an innovative and effective alternative approach to prevent p66Shc-mediated oxidative stress.

Noncoding RNAs (ncRNAs), which comprise various types of RNAs, including microRNAs (miRNAs), long ncRNAs (lncRNAs), and circular RNAs (circRNAs), have emerged as powerful gene expression regulators that play crucial roles in disease development 13. Among them, miRNAs represent a widespread class of ncRNAs that negatively regulate gene expression by hampering mRNA translation or favoring mRNA degradation 14. A previous study has shown that the miRNA let-7a regulates the lifespan of human diploid fibroblasts by repressing p66Shc expression 15. In addition, miR-27b can suppress p66Shc expression and mitochondrial oxidative stress, thus exerting protective effects against diabetes mellitus 16. However, it is not yet clear whether miRNAs participate in p66Shc regulation in the context of intestinal I/R injury.

Numerous studies have highlighted the important roles of ncRNA regulatory networks in the progression of human diseases. Several types of ncRNAs contain miRNA binding sites and function as competitive endogenous RNAs (ceRNAs), interacting with miRNAs to regulate target mRNA expression 17-19. CircRNAs, a novel type of ncRNA, are generated by back-splicing of single pre-mRNAs and are characterized as covalently closed loop structures 20. These endogenous ncRNAs display high tissue-specific expression and are generally more stable than their linear counterparts because they lack accessible ends and are thus resistant to exonucleases 21-23. Emerging evidence shows that circRNAs can act as powerful miRNA “sponges” to regulate gene expression. For example, ciRS-7 has more than 70 miR-7 binding sites and thus serves as an effective miR-7 suppressor to regulate miR-7 target mRNA expression 24. Recently, it has been reported that the circRNA MFACR mediates myocardial infarction by acting as a sponge of miR-652-3p to promote MTP18 expression 25. However, whether the circRNA-miRNA axis is involved in controlling p66Shc expression during intestinal I/R remains largely unknown.

In this study, we demonstrated that miR-339-5p expression, which was downregulated after intestinal I/R, was negatively correlated with p66Shc expression. Low miR-339-5p expression or high p66Shc expression was closely associated with intestinal epithelial oxidative stress, and overexpressed miR-339-5p significantly reduced ROS generation by targeting p66Shc. Furthermore, we identified a circRNA derived from the protein kinase C beta (PRKCB) gene locus, termed circ-PRKCB, that could bind directly to miR-339-5p and was upregulated after intestinal I/R. circ-PRKCB promoted intestinal I/R-induced ROS accumulation by sponging miR-339-5p and upregulating p66Shc expression. Our findings indicate that the circ-PRKCB/miR-339-5p/p66Shc axis, a regulator of oxidative stress in the intestine, is a potential therapeutic target for the treatment of intestinal I/R injury.

## Results

### p66Shc is a critical mediator of oxidative stress during intestinal I/R

**Figure 1:**
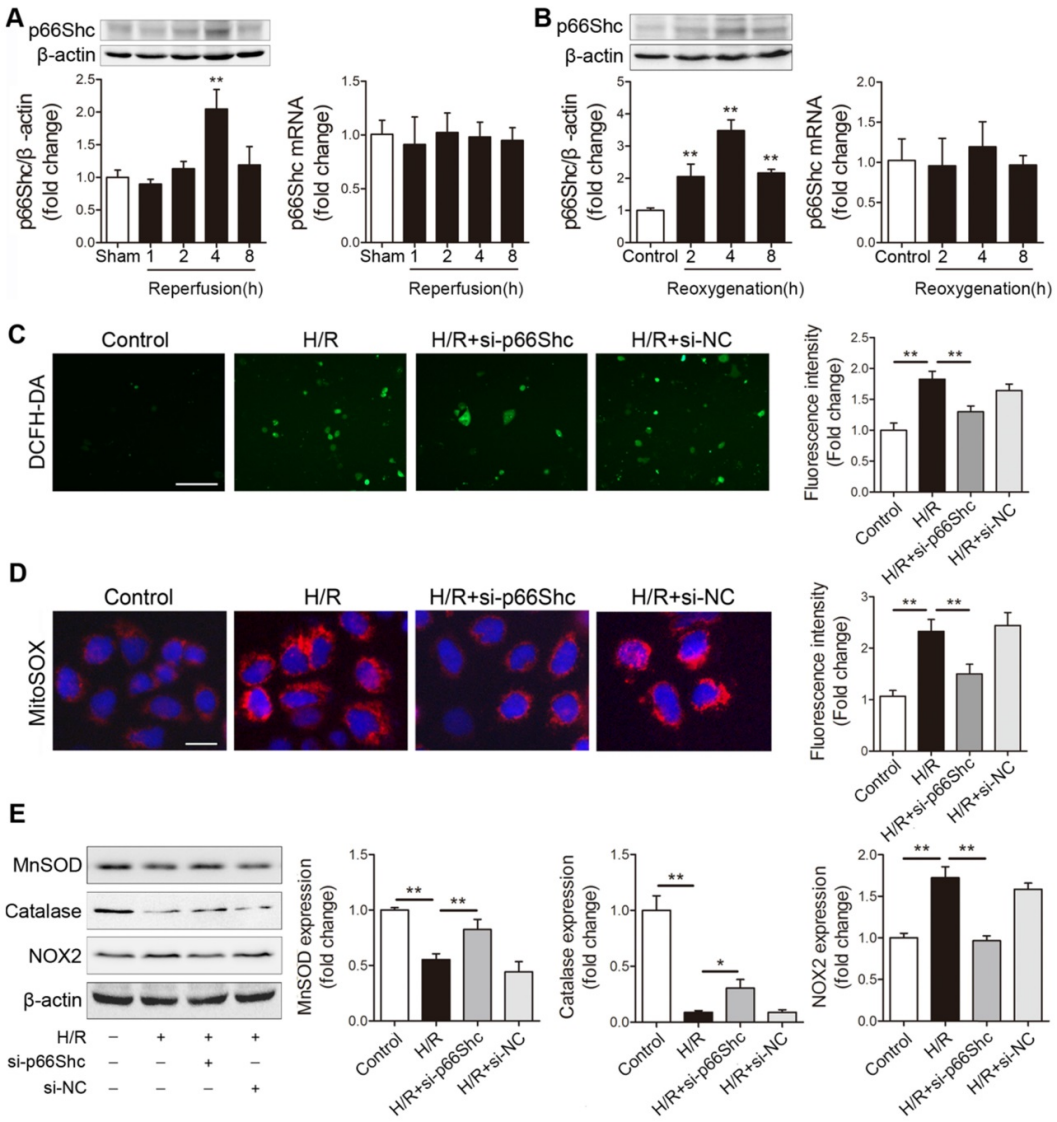
** p66Shc plays a crucial role in mediating ROS accumulation after intestinal I/R injury.** (A) Western blot and qRT-PCR analysis were used to assess p66Shc expression in the intestines of mice subjected to 45 min of intestinal ischemia followed by 1-8 h of reperfusion or to sham surgery (n=5). **P<0.01 compared with the sham group. (B) Western blot and qRT-PCR analysis were used to assess p66Shc expression in Caco-2 cells exposed to 15 h of hypoxia followed by 2-8 h of reoxygenation or to normoxic conditions (control) (n=5). **P<0.01 compared with the control group. (C-E) Caco-2 cells were transfected with si-p66Shc or si-NC and then subjected to hypoxic conditions for 15 h followed by normoxic conditions for 4 h to achieve H/R. (C, D) Representative fluorescence images and fluorescence quantification of DCFH-DA (C)- and MitoSOX (D)-stained cells. Scale bars, 100 µm (C) and 12.5 µm (D). (E) MnSOD, catalase and NOX2 protein expression (n=3). *P<0.05, **P<0.01. The values represent the mean ± SD.

Our previous work has revealed that p66Shc-mediated mitochondrial oxidative stress is critically involved in intestinal I/R injury 12. However, the mechanism by which p66Shc regulates intestinal I/R-induced ROS overproduction remains to be fully clarified. Here, we first detected p66Shc expression after intestinal I/R *in vivo* and *in vitro*. p66Shc protein levels increased in a reperfusion time-dependent manner in the mouse intestine (Figure 1A). Similarly, time-dependent increases in p66Shc levels were observed in human Caco-2 cells upon hypoxia/reoxygenation (H/R) treatment (Figure 1B). Intriguingly, intestinal I/R or H/R did not significantly influence p66Shc mRNA levels (Figure 1A-B). To further investigate the involvement of p66Shc in oxidative stress in intestinal epithelial cells, Caco-2 cells were transfected with p66Shc siRNA following H/R challenge. As expected, p66Shc silencing markedly decreased intracellular ROS levels upon H/R insult (Figure 1C). It has been reported that p66Shc sustains intracellular ROS concentrations in at least three ways: by catalyzing ROS formation from the mitochondrial respiratory chain, by activating membrane-bound NADPH oxidases and by downregulating antioxidant enzyme synthesis 26. Our data showed that compared with normoxia, H/R exposure markedly increased mitochondrial superoxide anion (O_2_^-^) levels and enhanced NOX2 (NADPH oxidasecore subunit) expression but decreased manganese superoxide dismutase (MnSOD) and catalase expression; these changes were significantly ameliorated after silencing of p66Shc (Figure 1D-E). Thus, these results indicate that p66Shc plays an important role in intestinal I/R-induced oxidative stress. The underlying mechanism by which p66Shc mediates ROS accumulation during intestinal I/R may be correlated with mitochondrial ROS promotion, NADPH oxidase activity enhancement, and antioxidant enzyme expression suppression.

### miR-339-5p is involved in regulating p66Shc expression in the intestine

**Figure 2:**
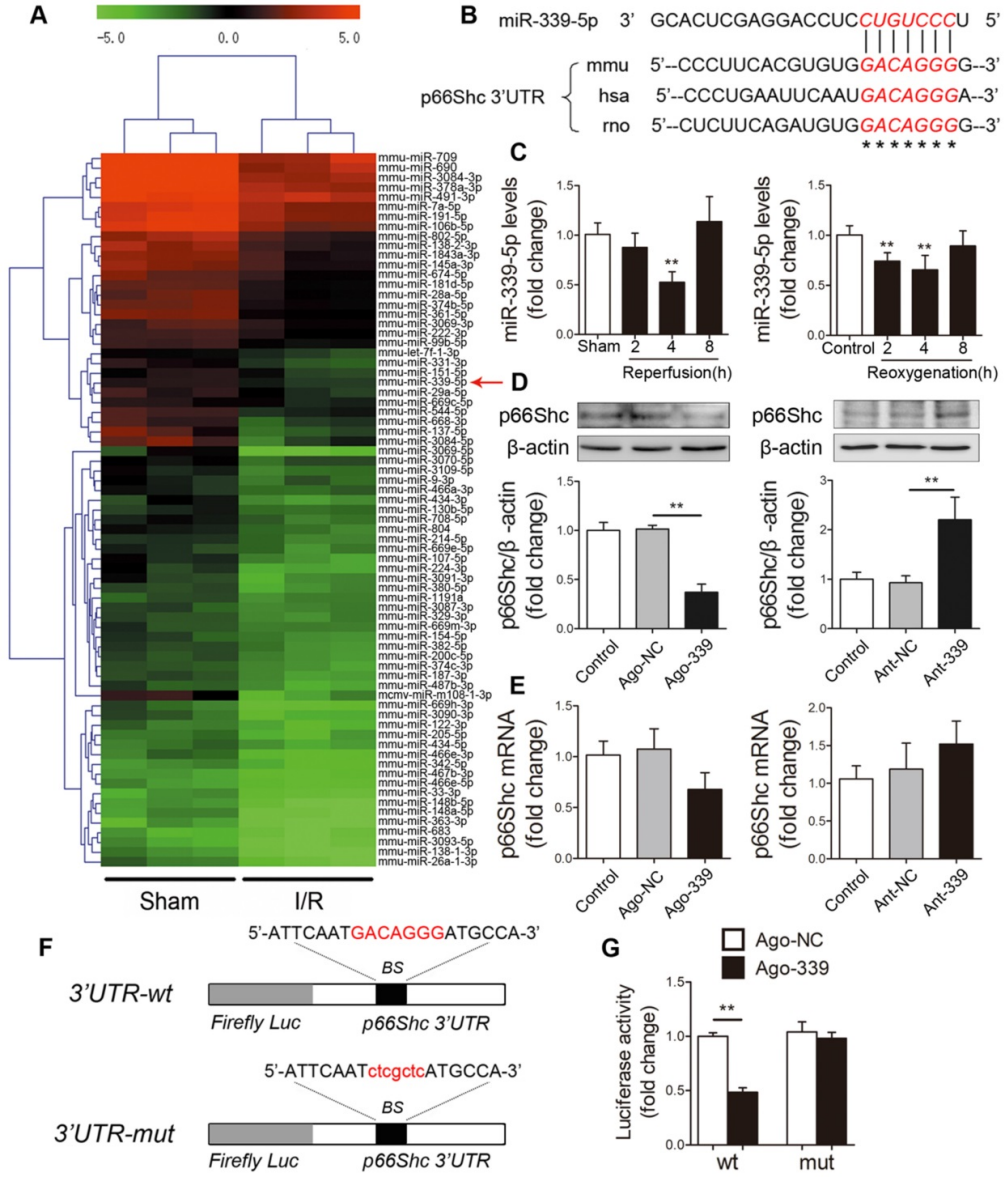
** miR-339-5p participates in regulating p66Shc expression.** (A) Hierarchical clustering analysis of 74 miRNAs that were significantly downregulated in intestinal I/R samples compared with sham samples (greater than 2.0-fold change, *P*< 0.05). The expression levels are represented with shades of red and green, which indicate expression above and below the median expression level across all samples (log_2_ scale, from -5.0 to +5.0), respectively. (B) A conserved binding site for miR-339-5p exists in the 3'UTR of p66Shc. (C) The expression levels of miR-339-5p in mouse I/R intestines (**P<0.01 compared with the sham group) (left) and H/R-treated Caco-2 cells (**P<0.01 compared with the control group) (right) were analyzed by qRT-PCR (n=6). (D-E) Caco-2 cells were transfected with ago-339, ant-339 or negative controls. (D) p66Shc protein expression (n=3). (E) p66Shc mRNA expression (n=6). (F) The miR-339-5p binding site was mutated in the p66Shc 3'UTR. BS, binding site. (G) Luciferase activity of luc-p66Shc-wt or luc-p66Shc-mut in Caco-2 cells cotransfected with ago-339 or ago-NC (n=6). **P<0.01. The values represent the mean ± SD.

Next, we explored the underlying mechanism by which p66Shc is upregulated upon I/R insult. The inconsistencies between mRNA and protein levels strongly suggested that a posttranscriptional mechanism may regulate p66Shc during intestinal I/R. Emerging evidence has revealed that miRNAs can regulate gene expression at the posttranscriptional level by binding to target mRNA molecules and inducing their degradation or inhibiting their translation. To investigate whether miRNAs regulate p66Shc in the context of intestinal I/R, we subjected mice to 45 min of intestinal ischemia followed by 4 h of reperfusion. Microarray analysis was then performed to determine the differentially expressed miRNA profiles in the murine intestine after I/R (GEO accession number: GSE83701) 27. We found that 57 miRNAs were upregulated and 74 miRNAs were downregulated (fold change≥2, *P*<0.05) in the I/R group compared with the sham group. We next focused on the 74 downregulated miRNAs, which we considered to be potential negative regulators of p66Shc expression during intestinal I/R (Figure 2A). According to the results of three computational algorithms, TargetScan, miRanda and RNAhybrid, 14 miRNAs were predicted to bind the 3' untranslated region (UTR) of p66Shc. However, only miR-339-5p and miR-342-5p had highly conserved binding sites in the 3'UTR of p66Shc. Our microarray analysis showed that miR-339-5p had a higher fold change and lower *P* value than miR-342-5p. Thus, we selected miR-339-5p for further experimental verification (Figure 2B). qRT-PCR assays confirmed that intestinal miR-339-5p expression progressively decreased after 2-4 h of reperfusion or reoxygenation. Thereafter, miR-339-5p expression recovered and reached nearly normal levels after 8 h of reperfusion or reoxygenation (Figure 2C). To test whether miR-339-5p is involved in regulating p66Shc expression, Caco-2 cells were transfected with a miR-339-5p agomir (ago-339) or antagomir (ant-339). As shown in Figure 2D, ago-339 significantly reduced p66Shc protein levels, whereas ant-339 markedly increased p66Shc protein levels. However, no apparent changes in p66Shc mRNA levels were observed in either group (Figure 2E). These data suggest that miR-339-5p has the potential to regulate intestinal p66Shc expression and that this regulation may involve repression of translation rather than effects on mRNA degradation. In addition, we constructed wild-type and mutant p66Shc 3'UTR luciferase reporters to further validate that miR-339-5p targets the 3'UTR of p66Shc (Figure 2F). The results indicated that miR-339-5p overexpression significantly impaired p66Shc 3'UTR luciferase activity but did not significantly affect mutant p66Shc 3'UTR luciferase activity (Figure 2G). These data suggest that miR-339-5p can bind directly to the p66Shc 3'UTR and regulate its expression in the intestine.

### miR-339-5p suppresses H/R-induced oxidative stress by targeting p66Shc *in vitro*

**Figure 3:**
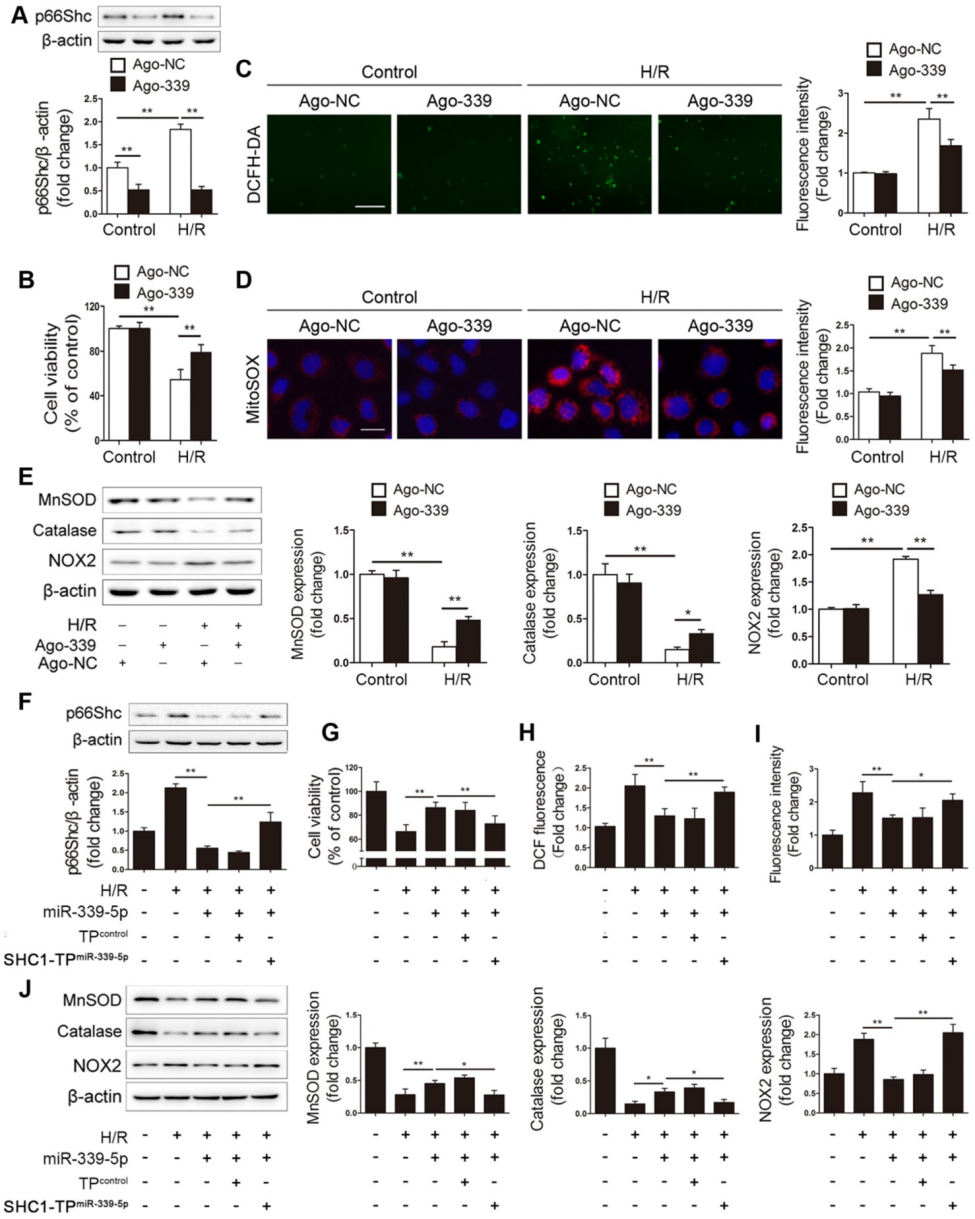
** miR-339-5p suppresses H/R-induced oxidative stress by targeting p66Shc *in vitro*.** (A-E) Caco-2 cells were transfected with ago-339 or ago-NC and then subjected to H/R. (A) p66Shc protein expression (n=3). (B) Cell viability (n=6). (C, D) Representative fluorescence images and fluorescence quantification of DCFH-DA (C)- and MitoSOX (D)-stained cells. Scale bars, 100 µm (C) and 12.5 µm (D). (E) MnSOD, catalase and NOX2 protein expression (n=3). (F-J) Ago-339 and SHC1-TP^miR-339-5p^ or a negative control were cotransfected into Caco-2 cells subjected to H/R. (F) p66Shc protein expression (n=3). (G) Cell viability (n=6). (H, I) Fluorescence quantification of DCFH-DA (H)- and MitoSOX (I)-stained cells. (J) MnSOD, catalase and NOX2 protein expression (n=3). *P<0.05, **P<0.01. The values represent the mean ± SD.

Having determined the regulatory effect of miR-339-5p on p66Shc expression, we next elucidated the functional role of miR-339-5p in intestinal epithelial oxidative stress. Ago-339 or an agomir negative control (ago-NC) was transfected into Caco-2 cells before H/R stimulation. As expected, forced miR-339-5p expression efficiently suppressed p66Shc expression and improved cell viability after H/R injury (Figure 3A-B). In addition, miR-339-5p overexpression significantly reduced intracellular ROS levels, mitochondrial O_2_^-^ levels and NOX2 expression and reversed H/R-induced MnSOD and catalase downregulation (Figure 3C-E). These data indicate that miR-339-5p acts as a negative regulator of oxidative stress and inhibits ROS overproduction induced by H/R.

To verify that the effect of miR-339-5p on oxidative stress is p66Shc dependent, a target protector technique was employed to disrupt the specific interactions between miR-339-5p and p66Shc in Caco-2 cells. As expected, the inhibitory effect of miR-339-5p on p66Shc expression was significantly abolished in the presence of the p66Shc target protector (Figure 3F). Moreover, the effects of miR-339-5p on cell viability, ROS levels, and NOX2, MnSOD and catalase expression were attenuated by the target protector under H/R (Figure 3G-J). Hence, p66Shc is specifically required for miR-339-5p-mediated protection against oxidative stress during intestinal H/R injury.

### miR-339-5p attenuates intestinal I/R injury *in vivo*

**Figure 4:**
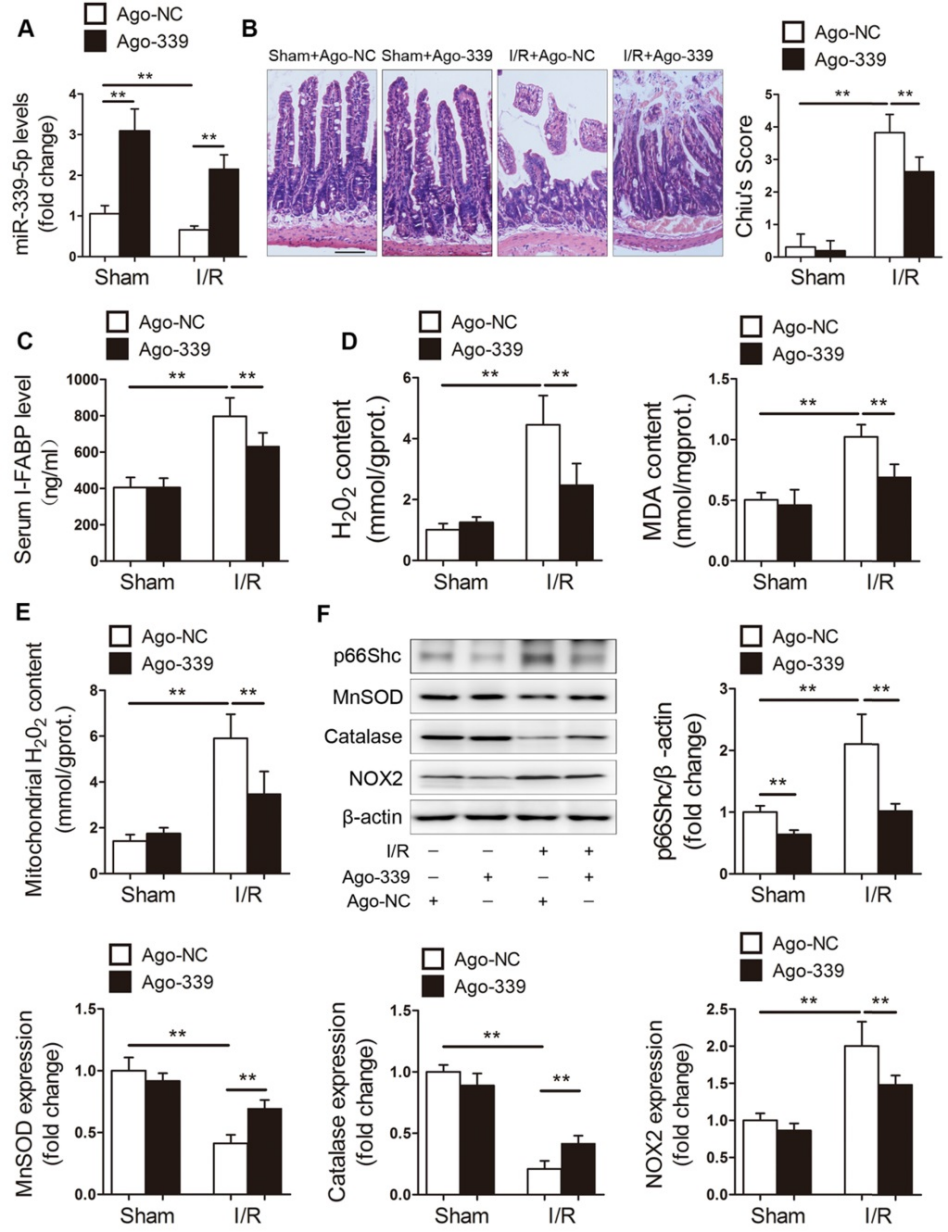
** miR-339-5p ameliorates intestinal I/R injury and oxidative stress *in vivo*.** C57BL/6 mice received tail vein injections of ago-339 or ago-NC for 3 consecutive days. They were then subjected to 45 min of intestinal ischemia followed by 4 h of reperfusion on the fourth day after injection. (A) miR-339-5p expression levels (n=8). (B) H&E staining and histological injury scoring (Chiu's score) of the intestinal mucosa. Scale bar, 100 µm (n=8). (C) Serum I-FABP levels (n=8). (D) H_2_O_2_ and MDA content in the intestine (n=8). (E) Mitochondrial H_2_O_2_ levels in the intestine (n=8). (F) p66Shc, MnSOD, catalase and NOX2 protein expression (n=3). *P<0.05, **P<0.01. The values represent the mean ± SD.

We next examined the role of miR-339-5p in intestinal I/R injury *in vivo*. Mice were administered ago-NC or ago-339 intravenously and subjected to intestinal I/R. As shown in Figure 4A, the miR-339-5p levels were decreased in the intestines of mice with I/R injury, while ago-339 efficiently replenished miR-339-5p levels under I/R conditions. Compared to ago-NC treatment, ago-339 treatment significantly alleviated gut damage induced by I/R, as indicated by improvements in histological injury and decreases in the circulating levels of intestinal fatty acid-binding protein (I-FABP) (Figure 4B-C). In addition, ago-339 significantly reduced I/R-induced oxidative stress in the intestine, as indicated by remarkable decreases in H_2_O_2_ and MDA levels (Figure 4D). Furthermore, ago-339 inhibited the upregulation of p66Shc and NOX2, blunted the generation of mitochondrial ROS and preserved the expression of the antioxidant enzymes MnSOD and catalase after intestinal I/R (Figure 4E-F). These results indicate that miR-339-5p exerts protective effects against intestinal I/R injury by inhibiting oxidative stress.

### circ-PRKCB sponges miR-339-5p in the intestine

**Figure 5:**
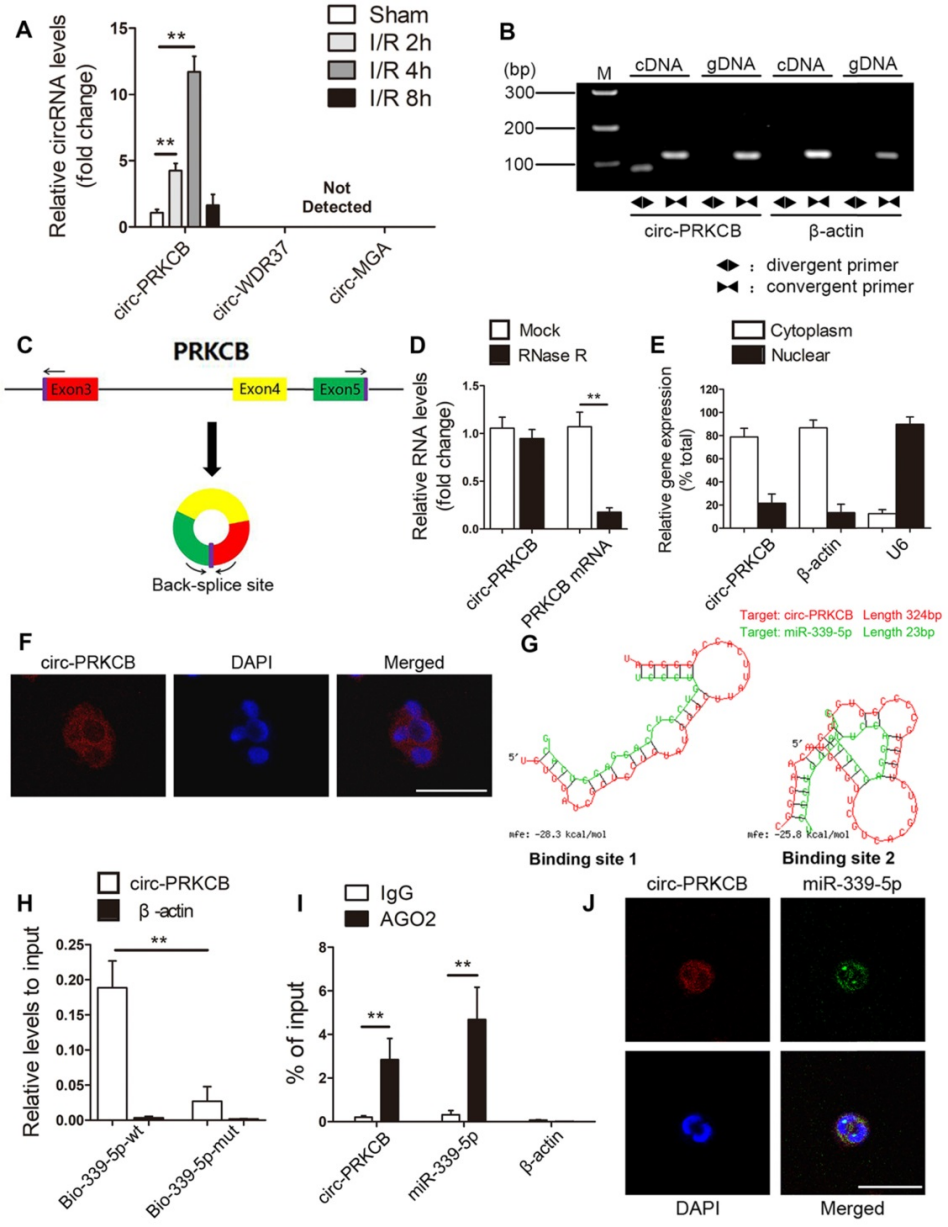
** circ-PRKCB binds to miR-339-5p.** (A) qRT-PCR analysis was used to determine circRNA expression levels in the intestines of mice subjected to 45 min of intestinal ischemia followed by 2-8 h of reperfusion or to sham surgery (n=5). (B) Divergent primers amplified circ-PRKCB in cDNA but not gDNA. β-actin, linear control. (C) Schematic diagram demonstrating that three exons derived from PRKCB constitute circ-PRKCB. (D) qRT-PCR analysis of circ-PRKCB expression after RNase R treatment in Caco-2 cells (n=6). (E) circ-PRKCB expression levels in the nuclear and cytoplasmic fractions of Caco-2 cells. β-actin and U6 were applied as positive controls for the cytoplasmic and nuclear fractions, respectively (n=6). (F) RNA-FISH indicated the location of circ-PRKCB in Caco-2 cells. The nuclei were stained with DAPI. Scale bar, 50 µm. (G) The bioinformatics program RNAhybrid showed detailed information for the two binding sites of miR-339-5p on circ-PRKCB. (H) Biotinylated wild-type miR-339-5p (Bio-339-5p-wt) or its mutant (Bio-339-5p-mut) was transfected into Caco-2 cells. After streptavidin capture, the circ-PRKCB levels were quantified by qRT-PCR, and the relative immunoprecipitate (IP)/input ratios were determined. β-actin was used as a negative control (n=3). (I) RIP analysis of circ-PRKCB, miR-339-5p and β-actin in Caco-2 cells using antibodies against AGO2. Each value was normalized to the level of input RNA used in the RIP analysis (n=3). (J) RNA-FISH indicated the colocalization between circ-PRKCB and miR-339-5p in Caco-2 cells. The nuclei were stained with DAPI. Scale bar, 50 µm. **P<0.01. The values represent the mean ± SD.

Emerging research findings have shown that circRNAs can function as miRNA sponges to regulate downstream targets. Consequently, we screened circRNAs that might act as sponges of miR-339-5p using the TargetScan, RNAhybrid, and miRanda tools. Considering sequence length and conservation between mice and humans, the three most promising candidates, circ-PRKCB (mmu_circ_0013842), circ-WDR37 (mmu_circ_0004848) and circ-MGA (mmu_circ_0001060), were identified. Then, qRT-PCR was performed to evaluate the expression of these predicted circRNAs in the mouse intestine in response to I/R treatment. As shown in Figure 5A, circ-PRKCB was progressively upregulated after 2-4 h of reperfusion. Thereafter, circ-PRKCB expression recovered and reached nearly normal levels after 8 h of reperfusion. However, circ-WDR37 and circ-MGA were not detected in the intestine. circ-PRKCB was amplified by divergent primers only from complementary DNA (cDNA); no amplification product was observed for genomic DNA (gDNA) (Figure 5B). circ-PRKCB was derived from exons 3, 4 and 5 of the PRKCB gene (Figure 5C). It was resistant to RNase R digestion, whereas linear PRKCB mRNA was markedly degraded (Figure 5D), confirming the circular nature of circ-PRKCB. We compared the sequences of circ-PRKCB in human and mice using the Basic Local Alignment Search Tool (BLAST) and found that the gene sequences of circ-PRKCB were highly conserved (Figure S1). In addition, qRT-PCR analysis of nuclear and cytoplasmic RNA and fluorescence *in situ* hybridization (FISH) revealed that circ-PRKCB was expressed mainly in the cytoplasm of Caco-2 cells (Figure 5E-F), suggesting that circ-PRKCB might regulate gene expression by acting as a miRNA sponge.

To test whether circ-PRKCB can bind miR-339-5p, we compared the sequences with the RNAhybrid bioinformatics program and identified two predicted binding sites (ΔG < -20kcal/mol) (Figure 5G). To further confirm the direct binding of miR-339-5p and circ-PRKCB, we carried out an RNA pull down assay using a biotin-coupled miR-339-5p mimic (Bio-339-5p-wt) and mutant (Bio-339-5p-mut). In Caco-2 cells transfected with biotinylated wild-type miR-339-5p, large amounts of circ-PRKCB were captured with miR-339-5p, while markedly decreased circ-PRKCB levels were observed in cells with mutated circ-PRKCB and miR-339-5p (Figure 5H). RNA immunoprecipitation (RIP) assays further showed that both circ-PRKCB and miR-339-5p were enriched in the Argonaute 2 (AGO2)-containing immunoprecipitates but not in the control IgG immunoprecipitates (Figure 5I).We also observed colocalization between circ-PRKCB and miR-339-5p in the cytoplasm via FISH analysis (Figure 5J). All these findings suggest that circ-PRKCB may function as a ceRNA to sponge miR-339-5p in the intestine.

### circ-PRKCB regulates p66Shc expression and H/R-induced oxidative stress by targeting miR-339-5p *in vitro*

**Figure 6:**
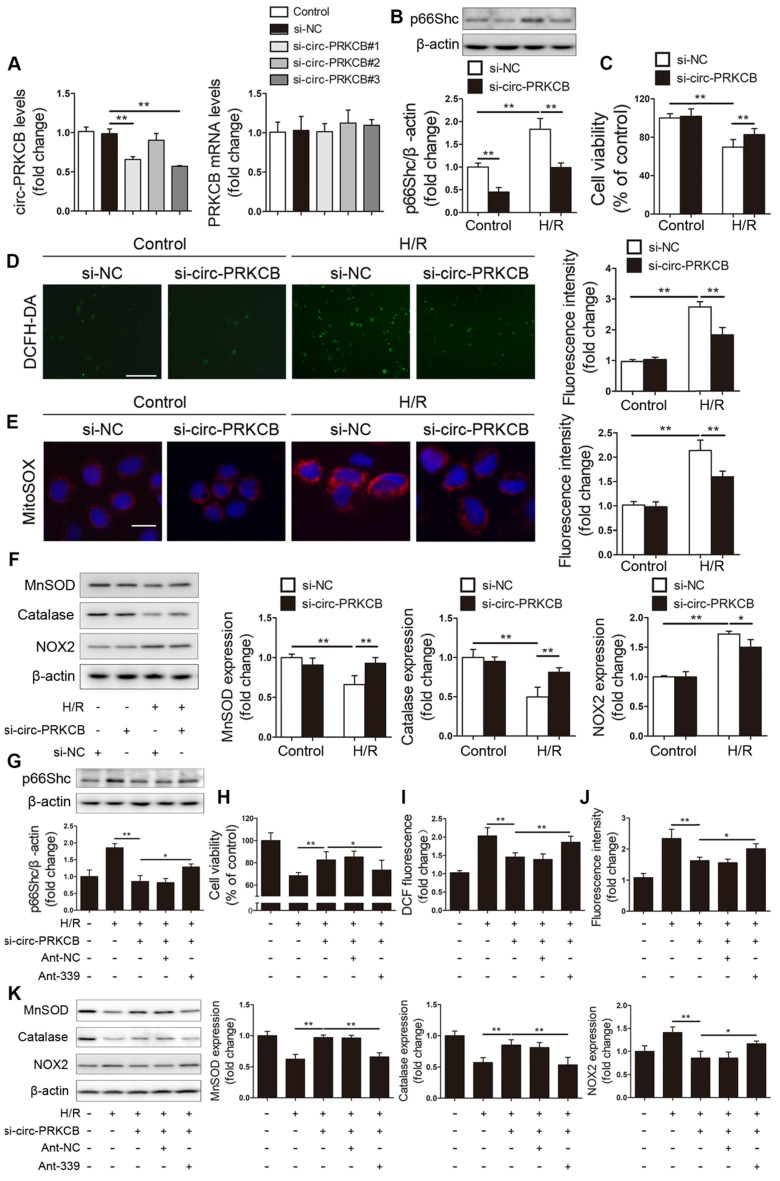
** circ-PRKCB regulates p66Shc expression and H/R-induced oxidative stress by targeting miR-339-5p.** (A) Caco-2 cells were transfected with si-circ-PRKCB or si-NC. qRT-PCR was conducted to detect circ-PRKCB and PRKCB mRNA expression (n=4). (B-F) Caco-2 cells were transfected with si-circ-PRKCB or si-NC and then subjected to H/R. (B) p66Shc protein expression (n=3). (C) Cell viability (n=6). (D, E) Representative fluorescence images and fluorescence quantification of DCFH-DA (D)- and MitoSOX (E)-stained cells. Scale bars, 100 µm (D) and 12.5 µm (E). (F) MnSOD, catalase and NOX2 protein expression (n=3). (G-K) Caco-2 cells were cotransfected with circ-PRKCB siRNA, ant-339 or ant-NC and then exposed to H/R. (G) p66Shc protein expression (n=3). (H) Cell viability (n=6). (I, J) Fluorescence quantification of DCFH-DA (I)- and MitoSOX (J)-stained cells. (K) MnSOD, catalase and NOX2 protein expression (n=3). *P<0.05, **P<0.01. The values represent the mean ± SD.

To investigate whether circ-PRKCB regulates p66Shc expression and H/R-induced oxidative stress by targeting miR-339-5p, we designed three different siRNAs for circ-PRKCB silencing (si-circ-PRKCB#1, si-circ-PRKCB#2 and si-circ-PRKCB#3); these siRNAs specifically targeted the back-splicing region of circ-PRKCB. We found that si-circ-PRKCB#1 and si-circ-PRKCB#3 successfully knocked down circ-PRKCB expression but had no effect on linear PRKCB mRNA expression in Caco-2 cells (Figure 6A). We selected si-circ-PRKCB#3 for further study because it had the best silencing efficiency. As shown in Figure 6B, silencing circ-PRKCB significantly suppressed p66Shc expression under both normoxic and H/R conditions. In addition, circ-PRKCB silencing markedly improved cell survival upon H/R insult (Figure 6C). Moreover, circ-PRKCB siRNA significantly inhibited the H/R-induced increases in intracellular ROS levels, mitochondrial O_2_^-^ levels and NOX2 expression and significantly ameliorated the H/R-induced decreases in MnSOD and catalase expression (Figure 6D-F).

To verify whether circ-PRKCB-mediated functional effects depend specifically on miR-339-5p/p66Shc axis, Caco-2 cells were cotransfected with circ-PRKCB siRNA, ant-339 or pcDNA-p66Shc. We found that ant-339 abolished the effect of circ-PRKCB silencing on p66Shc expression in Caco-2 cells under H/R (Figure 6G). Additionally, ant-339 eliminated the circ-PRKCB siRNA-mediated protection against H/R injury and oxidative stress (Figure 6H-K). Similarly, ectopic p66Shc expression could partially reverse the inhibitory effect of circ-PRKCB silencing on oxidative stress in Caco-2 cells under H/R (Figure S2). These results indicate that circ-PRKCB acts as an endogenous miR-339-5p sponge to regulate p66Shc expression and H/R-induced oxidative stress.

### Silencing circ-PRKCB protects the intestine from I/R injury *in vivo*

**Figure 7:**
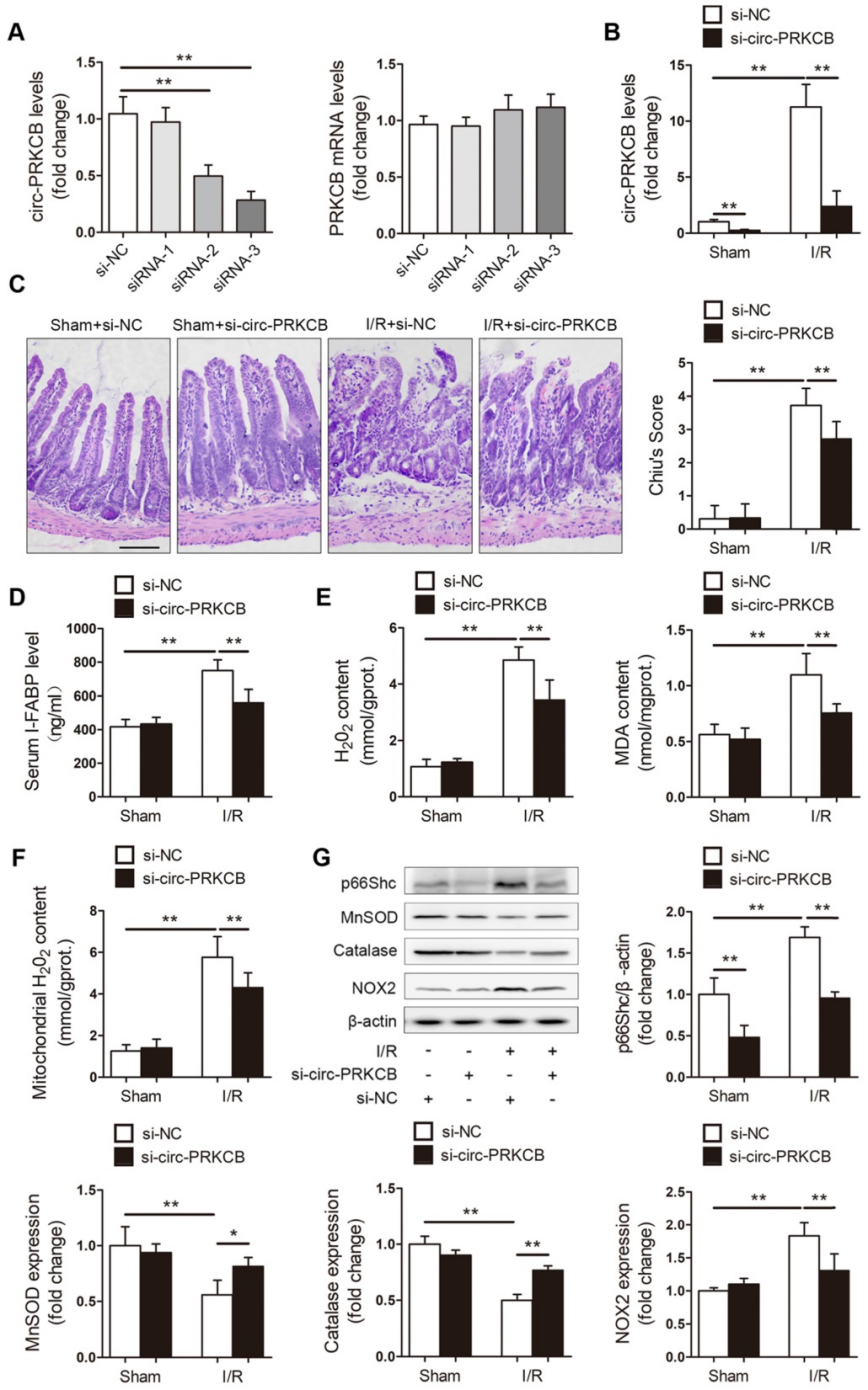
** Inhibition of circ-PRKCB attenuates intestinal oxidative damage induced by I/R.** (A) C57BL/6 mice were intravenously injected with si-circ-PRKCB or si-NC. 24 h after injection, qRT-PCR assays were conducted to determine circ-PRKCB and PRKCB mRNA expression (n=5). (B-G) C57BL/6 mice were intravenously injected with si-circ-PRKCB#3 or si-NC. They were then subjected to intestinal I/R 24 h after injection. (B) circ-PRKCB expression levels (n=8). (C) H&E staining and histological injury scoring (Chiu's score) of the intestinal mucosa. Scale bar, 100 µm (n=8). (D) Serum I-FABP levels (n=8). (E) H_2_O_2_ and MDA content in the intestine (n=8). (F) Mitochondrial H_2_O_2_ levels in the intestine (n=8). (G) p66Shc, MnSOD, catalase and NOX2 protein expression (n=3). *P<0.05, **P<0.01. The values represent the mean ± SD.

Next, we explored the effects of circ-PRKCB silencing on intestinal I/R injury in a mouse model. *In vivo* circ-PRKCB knockdown was performed by intravenous injection of 3 predesigned siRNAs specifically targeting the back-splicing sequence of circ-PRKCB. qRT-PCR was used to assessthe efficacy of the siRNAs in the mouse intestine. As shown in Figure 7A, circ-PRKCB siRNA-3 injection significantly reduced intestinal circ-PRKCB expression but not linear PRKCB mRNA expression and was thus selected for further study because of its silencing efficiency and specificity. Next, the mice were subjected to sham or I/R surgery. circ-PRKCB siRNA injection significantly attenuated the I/R-mediated increases in circ-PRKCB expression (Figure 7B). After intestinal I/R, the circ-PRKCB silencing group exhibited significantly milder histological injury and lower circulating I-FABP levels than the negative control group (Figure 7C-D). circ-PRKCB knockdown also attenuated the I/R-induced upregulation of H_2_O_2_ and MDA levels in the intestine (Figure 7E). Furthermore, our results showed that silencing circ-PRKCB significantly ameliorated I/R-induced intestinal oxidative stress, as revealed by decreases in mitochondrial ROS generation, reductions in p66Shc and NOX2 expression levels and increases in MnSOD and catalase expression levels in circ-PRKCB siRNA-injected mice (Figure 7F-G). Taken together, these results demonstrate that circ-PRKCB inhibition ameliorates intestinal oxidative damage induced by I/R in mice.

### circ-PRKCB/miR-339-5p/p66Shc signaling is dysregulated in the ischemic intestines of clinical patients

**Figure 8:**
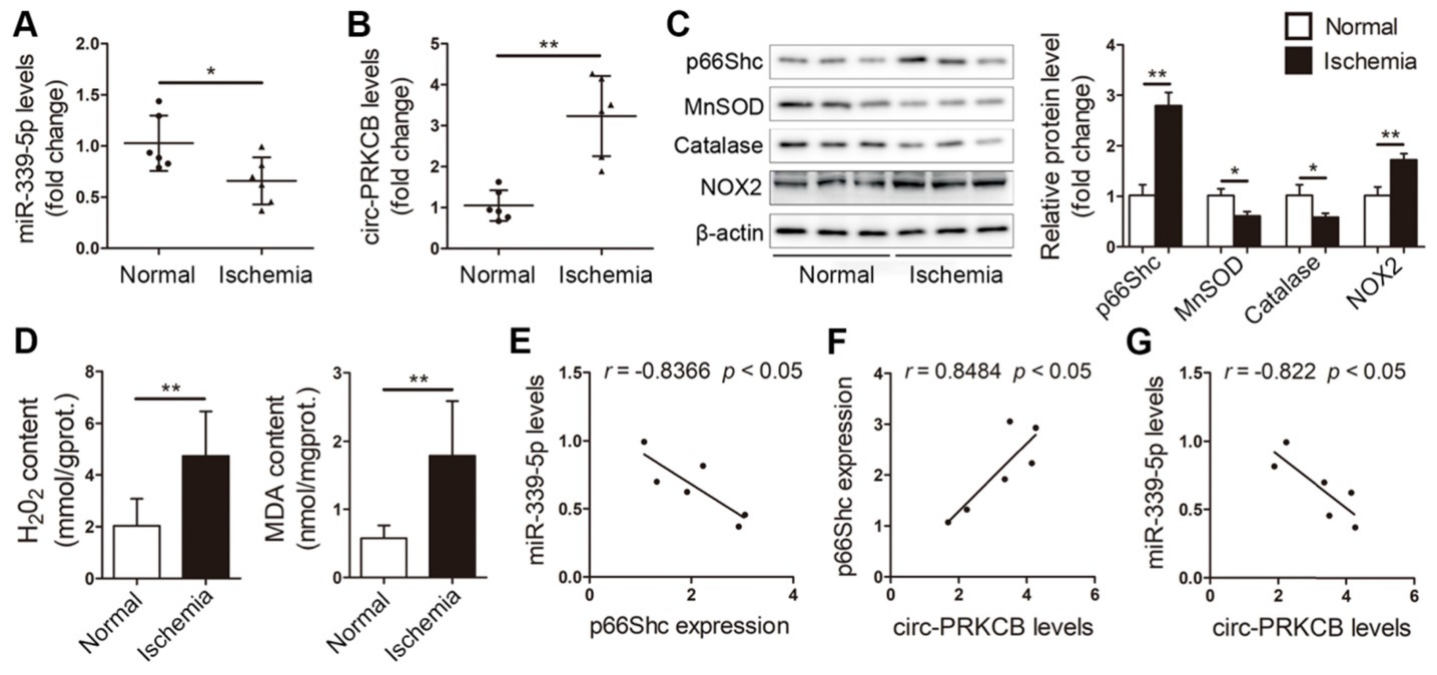
** circ-PRKCB/miR-339-5p/p66Shc signaling in ischemic intestines from clinical patients.** qRT-PCR analysis showing miR-339-5p expression (A) and circ-PRKCB expression (B) in human ischemic intestinal tissues and adjacent normal intestinal tissues (n=6). (C) p66Shc, MnSOD, catalase and NOX2 protein levels in human ischemic intestinal tissues and adjacent normal intestinal tissues (n=3). (D) H_2_O_2_ and MDA content in the intestine (n=6). (E) Correlation analysis between miR-339-5p and p66Shc expression in intestinal infarction samples (n=6). (F) Correlation analysis between circ-PRKCB and p66Shc expression in intestinal infarction samples (n=6). (G) Correlation analysis between miR-339-5p and circ-PRKCB expression in intestinal infarction samples (n=6). *P<0.05, **P<0.01. The values represent the mean ± SD.

We next assessed the clinical relevance of the circ-PRKCB/miR-339-5p/p66Shc signaling pathway in the intestinal mucosal tissues of patients suffering from intestinal infarction. qRT-PCR analysis showed that ischemic intestinal tissues exhibited lower miR-339-5p expression and higher circ-PRKCB expression than normal intestinal tissues from patients (Figure 8A-B). Consistently, p66Shc and NOX2 protein expression was significantly elevated, and antioxidant responses were dysregulated, as evidenced by decreases in MnSOD and catalase levels (Figure 8C). Furthermore, H_2_O_2_ and MDA levels were significantly elevated in ischemic intestinal tissues (Figure 8D). Intriguingly, Pearson correlation analysis showed a negative correlation between miR-339-5p and p66Shc expression and a positive correlation between circ-PRKCB and p66Shc expression in ischemic intestinal samples (Figure 8E-F). We also found that miR-339-5p expression was inversely correlated with circ-PRKCB expression in ischemic intestinal samples (Figure 8G). These results support the notion that the circ-PRKCB/miR-339-5p ceRNA regulatory axis could be a mechanism underlying the activation of p66Shc signaling in intestinal I/R injury.

## Discussion

In this study, we elucidated the involvement of the circ-PRKCB/miR-339-5p/p66Shc axis in intestinal I/R injury, particularly its involvement in oxidative stress. We made several observations. First, p66Shc is a critical mediator of oxidative damage during intestinal I/R. Specific knockdown of p66Shc significantly decreases ROS accumulation upon I/R insult. The underlying mechanism of intracellular ROS level reduction may be correlated with attenuation of mitochondrial oxidative stress, suppression of NADPH oxidase activity and enhancement of antioxidant enzyme expression. Second, a novel circRNA, circ-PRKCB, acts as a miR-339-5p sponge to participate in regulating p66Shc expression during intestinal I/R. Third, modulation of the circ-PRKCB/miR-339-5p axis can inhibit p66Shc expression, decrease p66Shc-mediated intestinal oxidative stress, and, as a result, alleviate intestinal I/R injury.

Our previous studies have shown that factors contributing to intestinal I/R injury are involved in multiple intracellular pathways, including oxidative stress, inflammation, apoptosis, ferroptosis and even autophagy 27-31. Moreover, uncontrolled activation of the innate immune system through toll-like receptors (TLRs) also plays an important role in I/R-mediated intestinal injury 32. In recent years, oxidative stress has emerged as the essential trigger in the pathogenesis of intestinal I/R. The oxygen influx that occurs during reperfusion contributes to redox imbalance in intestinal epithelial cells. ROS produced by various mechanisms can damage virtually all intracellular biomolecules, promote mitochondrial permeability transition pore opening, and activate necrotic and apoptotic cell death cascades to exacerbate cell injury. ROS-mediated mitochondrial damage also induces the release of damage-associated molecular pattern molecules, which can initiate epithelial immune hyper-responsiveness. This activation of the innate immune system causes inflammation. The above pathological factors eventually lead to intestinal epithelial cell dysfunction and destroy mucosal barrier integrity, thus aggravating reperfusion injury 1, 6, 33, 34. In animal experiments, exogenous ROS scavenger administration has been found to significantly reduce I/R-induced intestinal damage 35, 36, which suggests that interventions aimed at reducing ROS after intestinal I/R might be effective strategies for prevention of tissue injury. However, antioxidant treatment has failed to improve outcomes in previous large clinical trials 7. The reason may be related to the inability of exogenous antioxidants to significantly affect endogenous ROS levels. Hence, the development of treatment strategies such as preventing p66Shc activation to prevent increased ROS production, rather than lower it, may represent a more effective alternative.

The adaptor protein p66Shc is considered a key regulator of ROS production and a mediator of I/R damage in the intestine. However, the mechanisms by which p66Shc promotes intestinal oxidative stress induced by I/R remain to be fully clarified. Previous studies have reported that p66Shc modulates ROS production through at least three mechanisms. First, p66Shc promotes the assembly and activation of plasma membrane-bound NADPH oxidases 37, 38. Second, p66Shc downregulates antioxidant enzyme synthesis and suppresses ROS scavenging 39, 40. Third, p66Shc migrates into mitochondria, where it catalyzes electron transfer from cytochrome c to oxygen, resulting in mitochondrial ROS formation 41-43. We have previously demonstrated that intestinal I/R-induced ROS generation is due mainly to the catalysis of the mitochondrial respiratory chain and membrane-bound NADPH oxidases 29. Studies have also shown that intestinal I/R results in significant increases in mitochondrial ROS levels and NOX2 expression but decreases in antioxidant enzyme expression 31, 44, 45. In line with these observations, in the current study, we found that mitochondrial O_2_^-^ levels and NOX2 protein expression were significantly increased and that the expression levels of two important antioxidant enzymes, MnSOD and catalase, were significantly decreased upon H/R insult. However, these changes were significantly attenuated by p66Shc knockdown. Therefore, we suggest that p66Shc may increase intracellular ROS levels during intestinal I/R by catalyzing ROS formation in mitochondria, triggering of plasma membrane oxidases and suppression of antioxidant enzyme expression. The effects of p66Shc on other NOX family members or other enzymes needs to be further investigated in future studies.

MiRNAs have emerged as important regulators of gene expression at the posttranscriptional level. A recent report has shown that after 24 h of food deprivation, the protein levels of PR domain-containing 16 (PRDM16) are markedly decreased in the subcutaneous inguinal white adipose tissues of both male and female mice, whereas the mRNA levels of PRDM16 are not significantly changed. In addition, miR-149-3p has been identified to be involved in regulating PRDM16 expression in subcutaneous inguinal white adipose tissue 46. In this study, we also found inconsistent changes in p66Shc mRNA and protein levels after intestinal I/R. Therefore, we speculate that the expression levels of p66Shc may be regulated directly by certain miRNAs during intestinal I/R. Through a combination of microarray screening, bioinformatics analysis and qRT-PCR verification, we selected miR-339-5p for further exploration because it is a highly conserved p66Shc-targeting miRNA. Our subsequent gain- and loss-of-function experiments and a luciferase reporter assay showed that miR-339-5p targeted the p66Shc 3'UTR directly and repressed p66Shc expression both *in vivo* and *in vitro*, thereby leading to reduced oxidative stress levels in the context of intestinal I/R injury.

Recent studies have shown that circRNAs usually act as miRNA sponges and are involved in miRNA-mediated posttranscriptional regulation. This interaction greatly affects the expression of miRNA target genes. Increasing evidence suggests that circRNAs are key regulators of numerous biological functions during organ I/R injury. For example, circular RNA TLK1 functions as an endogenous miR-335-3p sponge to inhibit miR-335-3p activity, resulting in increases in TIPARP expression and subsequent exacerbation of neuronal injury and neurological deficits in the context of ischemic stroke 47. Similarly, another circRNA, circ-NCX1, mediates ischemic myocardial injury and promotes cardiomyocyte apoptosis by acting as an endogenous miR-133a-3p sponge and regulating CDIP1 expression 48. Consistent with the findings of previous studies, we identified a circRNA derived from the PRKCB gene locus, termed circ-PRKCB, as a potential miR-339-5p sponge. circ-PRKCB was localized mainly in the cytoplasm and was enriched in the AGO2 complex, the core subunit of the RNA-induced silencing complex (RISC), which is involved in miRNA-mediated gene repression. This result suggested that circ-PRKCB may act as a binding platform for AGO2 and miRNAs. Furthermore, the interaction between miR-339-5p and circ-PRKCB was confirmed via miRNA pull down and double FISH assays. All these findings suggest that circ-PRKCB exhibits ceRNA activity. We observed that circ-PRKCB and miR-339-5p were highly conserved between the mouse and human genomes. Moreover, we analyzed the complementarity of the circ-PRKCB sequence with miR-339-5p using the RNAhybrid bioinformatics program and found that the predicted miR-339-5p binding sites present within the circ-PRKCB sequence were also conserved between mice and human. Thus, it is possible that circ-PRKCB binds to miR-339-5p both *in vivo* and *in vitro*. Further functional experiments revealed that circ-PRKCB acts as a miR-339-5p sponge to regulate p66Shc expression and modulate intestinal oxidative stress induced by I/R. Although the present study shows that p66Shc is regulated by the circ-PRKCB/miR-339-5p axis during intestinal I/R, our results cannot rule out the possibility that other mechanisms may also contribute to p66Shc regulation. Therefore, further studies should determine whether mechanisms other than ceRNA activity control p66Shc expression to affect intestinal I/R-induced oxidative injury.

## Conclusions

In summary, the current study reveals, for the first time, the involvement of the novel upstream circ-PRKCB/miR-339-5p regulatory axis in the posttranscriptional regulation of p66Shc during oxidative stress in the context of I/R-induced intestinal injury. Our results elucidate the mechanism of the circ-PRKCB/miR-339-5p/p66Shc regulatory pathway, in which circ-PRKCB acts as a miR-339-5p sponge and inhibits the regulation of p66Shc by miR-339-5p. This study sheds light on a potential new method for targeted prevention of intestinal I/R-induced oxidative stress based on an ncRNA-based approach.

## Methods

### Animal experiments

Adult male C57BL/6 mice (8 weeks old) were obtained from the Laboratory Animal Center of Dalian Medical University (Dalian, China). All experiments were performed according to the Institutional Animal Care Guidelines and were approved by the Institutional Ethics Committee of Dalian Medical University. Intestinal I/R injury was surgically induced in mice as we have described previously 29. Briefly, the I/R mice were subjected to superior mesenteric artery occlusion with a microvascular clamp for 45 min followed by 1, 2, 4 or 8 h of reperfusion. The sham mice underwent the same procedure without vascular occlusion. The mice were sacrificed after the indicated period of reperfusion, and blood samples and terminal ileum tissues 5 cm away from the ileocecal valve were harvested for analysis.

To evaluate the effect of miR-339-5p on intestinal I/R injury, thirty-two mice were randomly divided into the following four groups: the sham+ago-NC group, the sham+ago-339 group, the I/R+ago-NC group, and the I/R+ago-339 group. The mice received intravenous injections of ago-339 or ago-NC at a dose of 35 mg/kg body weight/d for three consecutive days. Intestinal I/R was established on the fourth day. To evaluate the effect of circ-PRKCB knockdown on intestinal I/R injury, thirty-two mice were randomly divided into the following four groups: the sham+siRNA negative control (si-NC) group, the sham+circ-PRKCB siRNA (si-circ-PRKCB) group, the I/R+si-NC group, and the I/R+si-circ-PRKCB group. A commercially available cationic polymer transfection reagent (*in vivo*-jetPEI, Polyplus-transfection, Illkirch, France) was used to deliver the circ-PRKCB-targeting siRNAs via intravenous injection. Briefly, 100 µg of the selected siRNA was diluted in 200 µL of 5% glucose solution and mixed with the *in vivo*-jetPEI transfection reagent. The mixture was incubated for 15 min at room temperature to allow complexes to form. The mixture was then injected into the mice 24 h before surgery. The siRNAs and agomir were purchased from RiboBio (Guangzhou, China).

### Cell culture, H/R treatment and transfection

Caco-2 cells purchased from the American Type Culture Collection (ATCC, Manassas, VA, USA) were cultured in DMEM supplemented with 10% fetal bovine serum, 1% nonessential amino acids, 1% glutamine, and 1% penicillin/streptomycin and maintained in a humidified atmosphere with 5% CO_2_ at 37 °C. The Caco-2 cell H/R procedure was performed according to the methods in our previously published study 29. To mimic hypoxic conditions, the cells were incubated in a microaerophilic system (Thermo Fisher Scientific, Waltham, MA) with 5% CO_2_ and 1% O_2_ balanced with 94% N_2_ for 15 h. The cells were then cultured under normoxic conditions for 4 h to achieve reoxygenation.

The agomir, antagomir, p66Shc siRNA (si-p66Shc) and corresponding negative control (sequences listed in Table S1) were obtained from GenePharma (Shanghai, China). Three different siRNAs for circ-PRKCB silencing were provided by RiboBio. The p66Shc expression plasmid (pcDNA-p66Shc) and the control vector (pcDNA3.1) were synthesized by GenePharma. Cell transfection and cotransfection experiments were performed using Lipofectamine 3000 (Invitrogen, Shanghai, China) according to the manufacturer's protocols. After transfection for 48 h, the cells were challenged with H/R, and different assays were conducted.

### Patient tissue specimens

Terminal ileum samples were collected from six patients undergoing surgery for acute mesenteric arterial embolism, strangulated intestinal obstruction, or incarcerated hernia at the Second Hospital of Dalian Medical University (Dalian, China).Written informed consent was obtained from all the patients, and the process was approved by the Ethics Committee of Dalian Medical University.

### Intestinal histology

Intestinal tissues harvested from C57BL/6 mice were fixed in 4% paraformaldehyde and embedded in paraffin. Sections were stained with hematoxylin and eosin (H&E) and scored blindly for the severity and extent of intestinal lesions according to Chiu's scoring system 49.

### Mitochondrial isolation and H_2_O_2_, MDA and I-FABP examination

Mitochondria were isolated from intestinal samples using a mitochondria isolation kit (TransGen Biotech, Beijing, China) based on the manufacturer's instructions. The H_2_O_2_ and MDA levels in the intestine were determined using commercially available detection kits (Jiancheng Corp., Nanjing, China) according to the manufacturer's recommended protocol. The levels of serum I-FABP were determined with an enzyme-linked immunosorbent assay (ELISA) kit (R&D Systems) according to the manufacturer's instructions.

### Western blotting

Western blotting was conducted with primary antibodies against p66Shc (BD Biosciences, USA), MnSOD (Proteintech Group, Wuhan, China), catalase (Abcam Ltd., Cambridge, UK), NOX2 (Abcam Ltd.), and β-actin (ZSGB-BIO, Beijing, China). Protein quantification was performed using Gel-Pro Analyzer version 4.0 (Media Cybernetics, MD, USA).

### RNA extraction, RNase R treatment and qRT-PCR

Total RNA was isolated from tissues and cell lines with TRIzol reagent (Invitrogen, Carlsbad, CA, USA) according to the manufacturer's instructions. The nuclear and cytoplasmic fractions were extracted using a PARIS Kit (Invitrogen) following the manufacturer's instructions. gDNA was extracted using a Genomic DNA Isolation Kit (Takara, Dalian, China). RNase R treatment was conducted at 37 °C with 3 U/mg RNase R (Epicenter, WI, USA) for 15 min. cDNA was synthesized using a reverse transcription kit with PrimeScript^TM^ RT Master Mix (Takara). Real-time PCR analysis was carried out using a SYBR Premix Ex Taq^TM^ II kit (TaKaRa). circRNA and mRNA levels were normalized to β-actin levels. MiRNA levels were normalized to small nuclear U6 levels. The relative expression levels were determined by the 2^-ΔΔCt^ method. The details of the primers are listed in Table S1.

### Detection of intracellular and mitochondrial ROS levels

DCFH-DA (Sigma), a H_2_O_2_-sensitive fluorescence probe, was applied to assess intracellular H_2_O_2_ levels, as previously described. MitoSOX Red (Invitrogen), a live-cell-permeant dye that rapidly and selectively targets mitochondria, was used to observe mitochondrial O_2_^-^. Briefly, cells were loaded with 10 µM DCFH-DA for 30 min at 37 °C. The cells were then incubated with 5 µM MitoSOX Red for 10 min at 37 °C, and the nuclei were stained with Hoechst 33342 (Beyotime) for 5 min. The DCFH-DA fluorescence and MitoSOX Red fluorescence were visualized using a laser confocal microscope (LeicaTCS SP5II, Germany).

### Cell viability assay

Cell viability was measured by Cell Counting Kit-8 (CCK-8; Dojindo, Tokyo, Japan) assay according to the manufacturer's protocols. Briefly, after Caco-2 cells were seeded in 96-well plates and received appropriate treatments, 10 µl of CCK-8 reaction solution was added to each well at a 1/10 dilution. After 2 h of incubation at 37 °C, the absorbance of each individual well was measured at 450 nm by a microplate reader (Biotec, USA).

### RNA-FISH

circ-PRKCB and miR-339-5p probes were designed and synthesized by GenePharma. The signals of the probes were detected with a FISH kit (GenePharma) following the manufacturer's instructions. The specimens were analyzed with a Nikon inverted fluorescence microscope.

### Luciferase reporter assays

Plasmids containing wild-type or mutant miR-339-5p binding sites from the p66Shc 3′UTR were purchased from GenePharma. Plasmid DNA and ago-339 or ago-NC were cotransfected into Caco-2 cells seeded in 24-well plates using Lipofectamine 3000. The Caco-2 cells were evaluated with a Dual-Luciferase Reporter Assay Kit (TransGen) after 48 h of transfection. Luciferase activity was measured using a Dual-Light Chemiluminescent Reporter Gene Assay System (Berthold, Germany) and was normalized to Renilla luciferase activity.

### RIP

RIP experiments were performed with a Magna RIP RNA-Binding Protein Immunoprecipitation Kit (Millipore, Bedford, MA, USA) according to the manufacturer's instructions. An AGO2 antibody was used for RIP (Abcam). The coprecipitated RNA was detected by qRT-PCR.

### Biotin-coupled miRNA capture

A biotinylated miR-339-5p mimic and its mutant were synthesized by GenePharma. The sequence of the wild-type biotin-miR-339-5p mimic was 5'-bio-UCCCUGUCCUCCAGGAGCUCACG-3', while that of the mutant biotin-miR-339-5p mimic was 5'-bio-UGGGACAGCUCCAGGAGCUCACG-3'. A pull down assay was performed with the biotinylated miRNA as described in previous studies 50. The bound RNAs were purified using TRIzol reagent for further analysis.

### Statistical analysis

All values are expressed as the mean ± SD. Data with normal distributions were compared using one-way analysis of variance followed by the Student-Newman-Keuls test. A two-tailed Student's t-test was used to compare means between two groups. At least three independent experiments were performed to confirm the results. Statistical analysis was performed using GraphPad Prism 5.0 (GraphPad Software, CA, USA). A *p*-value < 0.05 was considered to indicate statistical significance.

## Supplementary Material

Supplementary figures and tables.

## Abbreviations

AGO2: Argonaute 2
circRNA: circular RNA
ceRNA: competitive endogenous RNA
DMEM: Dulbecco's modified Eagle's medium
ELISA: enzyme-linked immunosorbent assay
FBS: fetal bovine serum
FISH: fluorescence *in situ* hybridization
H&E: hematoxylin and eosin
H_2_O_2_: hydrogen peroxide
H/R: hypoxia/reoxygenation
I-FABP: intestinal fatty acid-binding protein
IP: immunoprecipitation
I/R: ischemia/reperfusion
lncRNA: long noncoding RNA
miRNA: microRNA
MnSOD: manganese superoxide dismutase
ncRNA: noncoding RNA
p66Shc: 66 kDa isoform of the adaptor molecule ShcA
PRKCB: protein kinase C beta
pre-mRNA: precursor mRNA
qRT-PCR: quantitative real-time polymerase chain reaction
RISC: RNA-induced silencing complex
ROS: reactive oxygen species
siRNA: small interfering RNA
SMA: superior mesenteric artery
UTR: untranslated region
