# Supplementary material for: circ-PRKCB acts as a ceRNA to regulate p66Shc-mediated oxidative stress in intestinal ischemia/reperfusion
Source: Theranostics. 2020 Aug 29;10(23):10680–96. doi: 10.7150/thno.44250 (PMC7482802; doi:10.7150/thno.44250)
Supplement: Supplementary file 1 — Supplementary figures and tables. [file thnov10p10680s1.pdf]

Supplementary Table and Figures

Table S1. PCR primer sequence, siRNA sequence, Agomirs and Antagomirs sequence

**Name**	**Sequences (5'-3')**
p66Shc mRNA for mouse	Forward: GTCCGACTACCTGTGTTCCTT<br>Reverse: CAGCAGGATTGGCCAGCTT
p66Shc mRNA for human	Forward: GAGGCCATCAGTCTGGTGTGT<br>Reverse: GCTGGTGGAGACGGTGAGA
PRKCB mRNA for mouse	Forward/convergent F: GCCATGAGTTCGTCACGTTC<br>Reverse/convergent R: TGAGAACCTCTCTGTCGATGTG
PRKCB mRNA for human	Forward: ATCGCCCCCGAGATAATTGC<br>Reverse: GGATGGCGGGTGAAAAATCG
β-actin mRNA for mouse	Forward/convergent F: AGAGGGAAATCGTGCGTGAC<br>Reverse/convergent R: CAATAGTGATGACCTGGCCGT
β-actin mRNA for mouse	Forward/ divergent F: ACGGCCAGGTCATCACTATTG<br>Reverse/ divergent R: GTCACGCACGATTTCCCTCT
β-actin mRNA for human	Forward: ACCCTGAAGTACCCCATCGAG<br>Reverse: ACATGATCTGGGTCATCTTCTCG
circ-PRKCB for mouse	Forward/ divergent F: CACATCGACAGAGAGGTTCTCA<br>Reverse/ divergent R: GAACGTGACGAACTCATGGC
circ-WDR37 for mouse	Forward: GACAGAACTGCCAACCTGTATG<br>Reverse: GGAAAACATTCACGGAGTGG
circ-MGA for mouse	Forward: CCAAAATCAAGGGATGGAGAG<br>Reverse: TTAAGATCAATGCTCACTGTTGG
circ-PRKCB for human	Forward: GCCGCATCTACATCCAGG<br>Reverse: TGCACCACAAAGCAGCAA
p66Shc siRNA	AUGAGUCUCUGUCAUCGCUTT<br>AGCGAUGACAGAGACUCAUTT
negative control siRNA	UUCUCCGAACGUGUCACGUTT<br>ACGUGACACGUUCGGAGAATT
circ-PRKCB siRNAs	Provided by Ribobio
miR-339-5p agomir	UCCCUGUCCUCCAGGAGCUCACG<br>UGAGCUCCUGGAGGACAGGGAUU
negative control agomir	UUCUCCGAACGUGUCACGUTT<br>ACGUGACACGUUCGGAGAATT
miR-339-5p antagomir	CGUGAGCUCCUGGAGGACAGGGA
negative control antagomir	CAGUACUUUUGUGUAGUACAA

mmu_circ_0013842|ENSMUST00000143692|Prkcb

Sequence ID: Query_54737 Length: 324 Number of Matches: 1

Range 1: 3 to 319

Score	Expect	Identities	Gaps
398 bits(215)	1e-115	283/317(89%)	0/317(0%)

```
Mouse  3    TGCTGCTTTGTGGTGCACAAGCGGTGCCATGAATTTGTCACATTCTCCTGCCCTGGCGCT  62
            ||||||||||| || |||||||| |||||||| || ||||| ||||||||||| || || 
Human  3    TGCTGCTTTGTTGTACACAAGCGCTGCCATGAGTTCGTCACGTTCTCCTGCCCCGGTGCG  62

Mouse  63   GACAAGGGTCCAGCCTCCGATGACCCCCGCAGCAAACACAAGTTTAAGATCCACACGTAC  122
            |||||||| || ||||| ||||||||||| ||||||||||||||||||||||||||||||
Human  63   GACAAGGGCCCGGCCTCTGATGACCCCCGGAGCAAACACAAGTTTAAGATCCACACGTAC  122

Mouse  123  TCCAGCCCCACGTTTTGTGACCACTGTGGGTCACTGCTGTATGGACTCATCCACCAGGGG  182
            ||||| || || || |||||||||||||| || |||||||||||||| || |||||||||
Human  123  TCCAGTCCTACCTTCTGTGACCACTGTGGATCGCTGCTGTATGGACTTATTCACCAGGGG  182

Mouse  183  ATGAAATGTGACACCTGCATGATGAATGTGCACAAGCGCTGCGTGATGAATGTTCCCAGC  242
            |||||||| |||||||| |||||||| ||||||||||||||||||||||| || ||||||
Human  183  ATGAAATGCGACACCTGTATGATGAACGTGCACAAGCGCTGCGTGATGAACGTCCCCAGC  242

Mouse  243  CTGTGTGGCACGGACCACACGGAGCGCCGCGGCCGCATCTACATCCAGGCCCACATCGAC  302
            || || ||||| ||||||||||| ||||||||||||||||| ||||||||||||||||||
Human  243  CTCTGCGGCACTGACCACACGGAACGCCGCGGCCGCATCTATATCCAGGCCCACATCGAC  302

Mouse  303  AGGGACGTCCTCATTGT  319
            || || || ||||||||
Human  303  AGAGAGGTTCTCATTGT  319
```

Figure S1. circ-PRKCB gene homology comparison.

circ-PRKCB homology between the human and mouse genomes was analyzed using the BLAST.

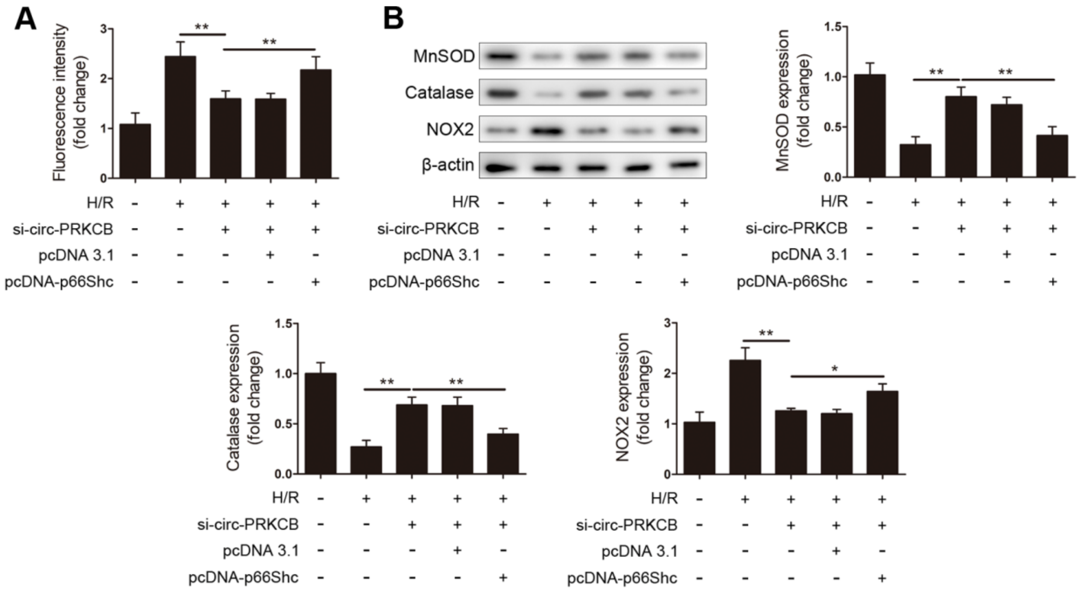

Figure S2. circ-PRKCB regulates H/R-induced oxidative stress through targeting p66Shc.

Caco-2 cells were cotransfected with circ-PRKCB siRNA or pcDNA-p66Shc and then exposed to H/R. **(A)** Fluorescence quantification of MitoSOX-stained cells. **(B)** MnSOD, catalase and NOX2 protein expression (n=3). *P<0.05, **P<0.01. The values represent the mean ± SD.
